# *NKAIN2* functions as a novel tumor suppressor in prostate cancer

**DOI:** 10.18632/oncotarget.11690

**Published:** 2016-08-30

**Authors:** Xueying Mao, Fei Luo, Lara K. Boyd, Bowei Zhou, Yanling Zhang, Elzbieta Stankiewicz, Jacek Marzec, Natasa Vasiljevic, Yongwei Yu, Ninghan Feng, Jia Xu, Attila Lorincz, Yong Jiang, Claude Chelala, Guoping Ren, Daniel M Berney, Shan-Chao Zhao, Yong-Jie Lu

**Affiliations:** ^1^ Centre for Molecular Oncology, Barts Cancer Institute, Barts and the London School of Medicine and Dentistry, Queen Mary University of London, London, EC1M 6BQ, UK; ^2^ Department of Urology, Nanfang Hospital, Southern Medical University, Guangzhou, 510515, China; ^3^ Key Laboratory of Proteomics of Guangdong Province and Key Laboratory of Transcriptomics and Proteomics of Human Diseases Supported by The Ministry of Education of China, Southern Medical University, Guangzhou, 510515, China; ^4^ Department of Pathology, The First Affiliated Hospital, Zhejiang University Medical College, Hangzhou, 310009, China; ^5^ Department of Gynecology and Obstetrics, Sir Run Run Shaw Hospital, Zhejiang University Medical College, Hangzhou, 310009, China; ^6^ Centre for Cancer Prevention, Wolfson Institute of Preventive Medicine, Barts and the London School of Medicine and Dentistry, Queen Mary University of London, London, EC1M 6BQ, UK; ^7^ Department of Pathology, Changhai Hospital, The Second Military Medical University, Shanghai, 200433, China; ^8^ Department of Urology, Wuxi Second People's Hospital, Nanjing Medical University, Wuxi, 214002, China

**Keywords:** NKAIN2, tumor suppressor, prostate cancer, chromosomal deletion and truncation, population difference

## Abstract

Recurrent chromosome breakpoints at 6q22.31, leading to truncation and potential loss-of-function of the *NKAIN2* gene, in Chinese prostate cancer patients were previously identified. In this study we investigated genomic, methylation and expression changes of *NKAIN2* in a large number of prostate cancer samples and determined its functional role in prostate cancer cells. Fluorescence *in situ* hybridization analysis confirmed that *NKAIN2* truncation is specific to Chinese while deletion of the gene is frequent in both Chinese and UK prostate cancers. Significantly reduced expression of *NKAIN2* was also detected at both RNA and protein levels. Somatic mutations of *NKAIN2* in prostate cancer samples exist but at very low frequency, suggesting that it is a putative tumor suppressor gene (TSG) with haploid insufficiency. Our functional studies showed that overexpression of *NKAIN2* in prostate cancer cells inhibits cellular growth by promoting cell apoptosis, and decreasing cell migration and invasion. Conversely, knockdown of *NKAIN2* promotes prostate cancer cell growth by inhibiting cell apoptosis, and increasing cell migration and invasion. These data imply that *NKAIN2* is a novel TSG whose activity is commonly reduced in prostate cancer. It may restrain the disease development and progression by inducing apoptosis and suppressing cancer cell growth, migration and invasion. This study provides new insights into prostate carcinogenesis and opportunities for development of novel therapies for prostate cancer.

## INTRODUCTION

Prostate cancer is the second leading cause of cancer death in Western men [1], accounting for 26% of new cases and 9% of deaths in the USA in 2015 [2]. Its prevalence is much higher in most Western populations than Asian countries, where prostate cancer studies are limited [1, 3]. Recently, we identified certain genomic alterations that differ between prostate cancer in Western and Chinese populations [4–6]. Further investigation of prostate cancer in Chinese men should lead to identification of novel genomic alterations that contribute to prostate cancer development and/or progression.

Our high-density SNP array analysis of prostate cancer samples collected from the UK and China revealed that chromosomal truncations frequently occur at known tumor suppressor genes (TSGs). We also identified recurrent chromosomal breakpoints within the Na^+^/K^+^ transporting ATPase interacting 2 (*NKAIN2*) gene located at 6q22.31 [7], which result in deletion of large portions of this gene. Given that 6q deletions covering this genomic region are common in many human cancers, including prostate cancer [4, 8, 9], *NKAIN2* is a potential TSG. Interestingly, chromosomal truncations disrupting *NKAIN2* were observed in four of 39 Chinese samples and only in one of 32 UK samples [7], suggesting that *NKAIN2* truncation may be more prone to occur in the Chinese population. However, this has yet to be validated in an additional series of samples. Therefore, we further screened larger cohorts of Chinese and UK prostate samples to evaluate the frequency of *NKAIN2* truncation and deletion, as well as its expression changes, and investigated its cellular function in association with tumorigenesis *in vitro*. We confirmed that *NKAIN2* is a frequently down-regulated gene in prostate cancer, particularly in Chinese cases, and found that it suppresses prostate cancer cell growth and invasion, suggesting its role as a novel putative TSG.

## RESULTS

### Confirmation of prevalence of *NAKIN2* genomic alterations in prostate cancer

**Figure 1:**
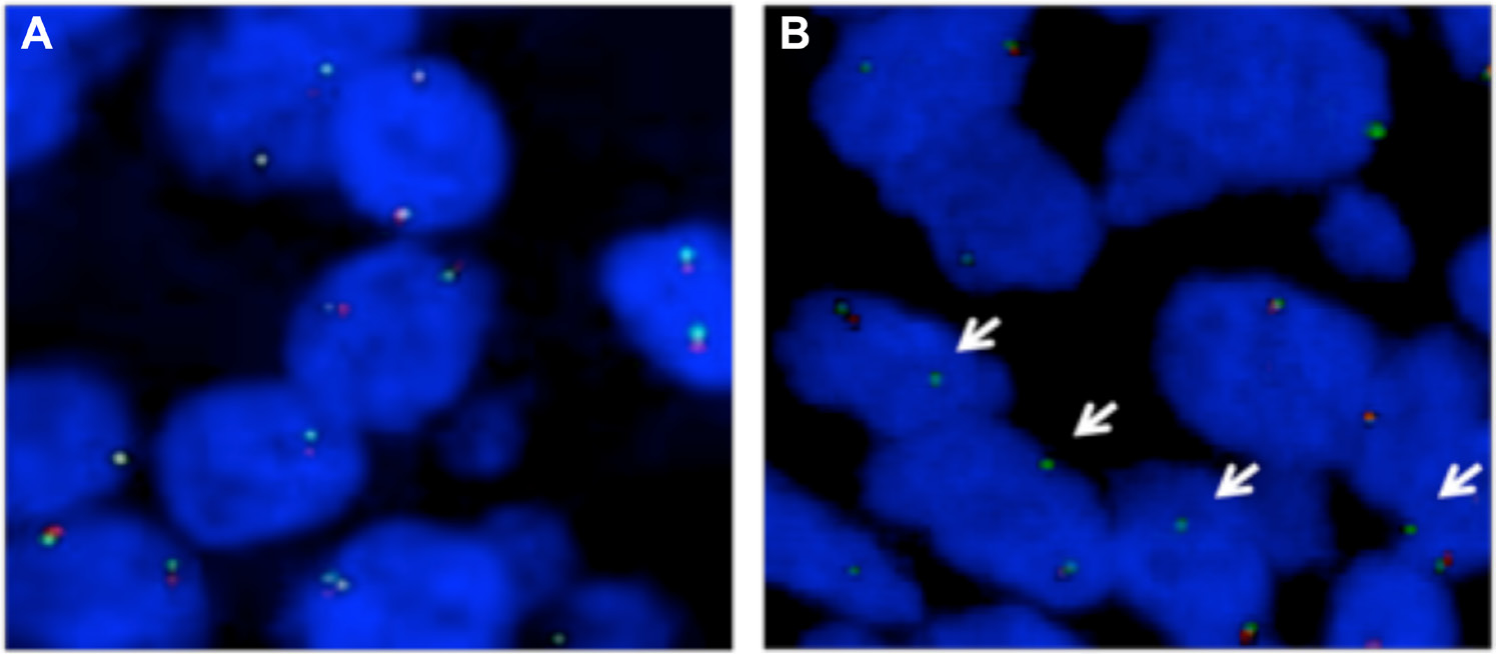
Fluorescence *in situ* hybridization (FISH) analysis on tissue microarrays to confirm recurrent *NKAIN2* breakpoints in prostate cancer (**A**) Representative FISH image showing a UK case in which normal copy number of *NKAIN2* was detected without breakpoint (two pairs of red and green signals). (**B**) Representative FISH image showing a Chinese case with *NKAIN2* breakpoint indicated by the loss of red signal (arrows).

To further assess the frequency of genomic alterations of *NKAIN2*, fluorescence *in situ* hybridization (FISH) analysis was performed on tissue microarrays (TMAs) containing 318 prostate cancer samples, including 194 cases from China and 124 cases from UK. Loss of either 5′ or 3′ FISH signal indicated a *NKAIN2* truncation event, and loss of FISH signal for both probes indicated a *NKAIN2* deletion event. *NKAIN2* truncation and deletion events were detected in 9/140 (6.4%) and 46/134 (34.3%) Chinese prostate cancer cases, respectively. split signals were not detected in any of the 103 UK cases for which FISH signals could be counted (Figure 1) confirming that the *NKAIN2* gene is more frequently truncated in Chinese than UK prostate cancers (*p* = 0.01). Consistent with our earlier study, *NKAIN2* gene deletion events were detected at a similar frequency in prostate cancer cases taken from the UK (21/89, 23.2%) and China (no statistical significance: *p* = 0.10).

### *NKAIN2* expression is reduced in prostate cancer

**Figure 2:**
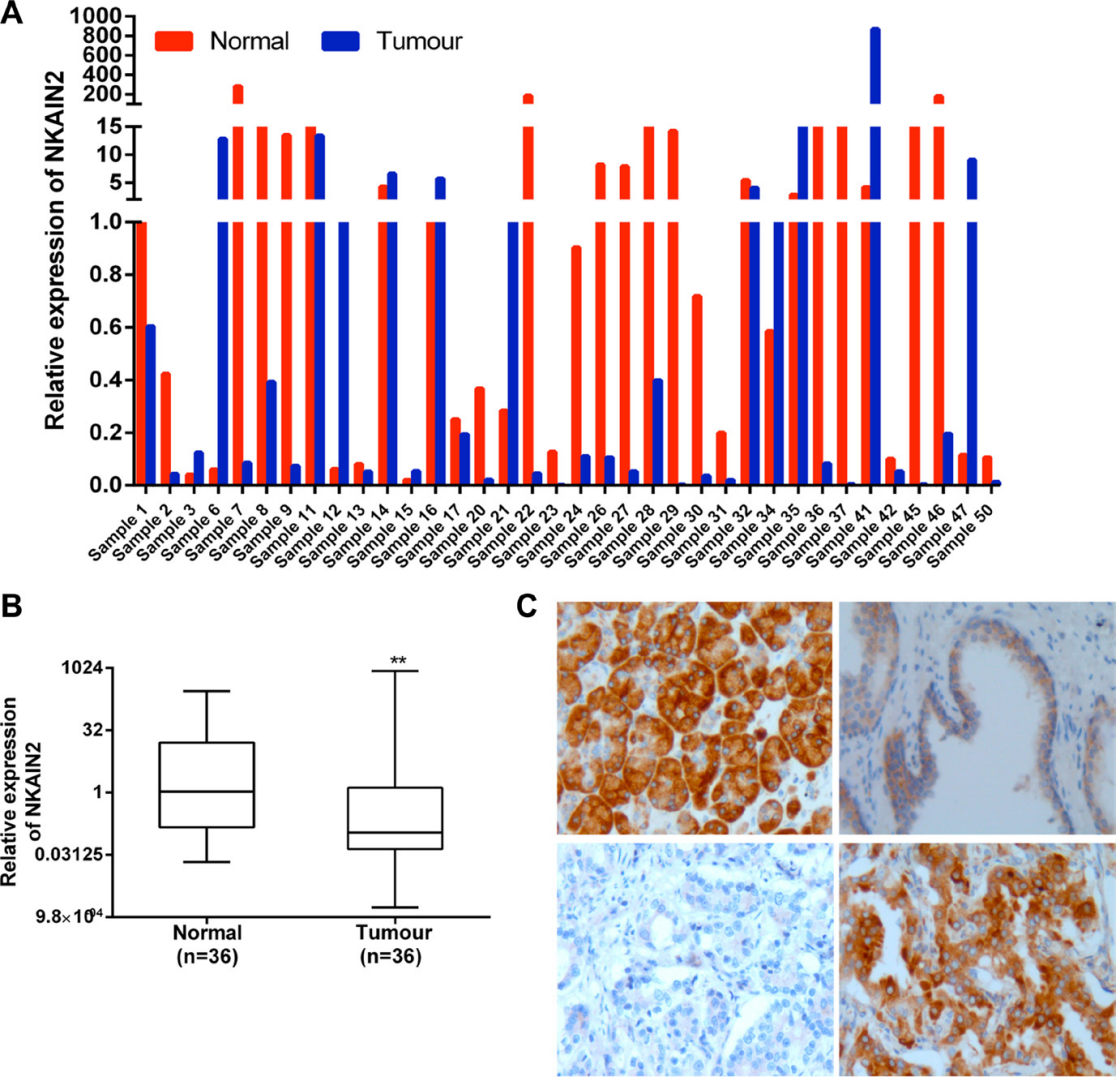
*NKAIN2* expression is commonly decreased in prostate cancer (**A**) Bar chart showing relative *NKAIN2* RNA expression in each of the 36 cases of tumor and matched normal prostate cancer tissues from China. (**B**) Relative RNA expression of *NKAIN2* compared to corresponding normal prostate tissues normalized to normal control sample 1. **p < 0.01. (**C**) Representative immunohistochemistry images for NKAIN2 protein expression in prostate cancer and adjacent normal tissues. Upper left: Strong immunostaining of the positive control tissue (pancreatic cancer); Upper right: Intermediate immunostaining (++) of the prostate epithelial cells in normal prostate gland and lack of staining in the prostate stromal cells; lower left: Lack of staining in the prostate cancer cells (−); Lower right: Strong staining of the prostate cancer cells (+++). All images were captured under the 20× objective lens.

mRNA expression of *NKAIN2* was analyzed by QRT-PCR in 36 paired tumor and adjacent normal prostate tissue samples from Chinese patients. *NKAIN2* expression was reduced in 25 cancer cases when compared to matched normal controls (Figure 2A), and overall expression was significantly lower in cancer than in the adjacent normal samples (*p* = 0.002)(Figure 2B). Subsequent examination of NKAIN2 protein expression by immunohistochemistry in 281 prostate cancer cases, including 161 cases from China and 120 cases from UK, revealed significant under-expression when compared to the adjacent normal tissues (*p* < 0.0001)(Table 1). All UK and most Chinese (97.3%) normal samples were positively stained, whereas 17.5% and 28% of the cancer samples from UK and China, respectively, were negative (Table 1)(Figure 2C).

**Table 1:** NKAIN2 immunohistochemistry results of prostate cancer cases from UK and China

	UK					China				
	–	+	++	+++	Total	–	+	++	+++	Total
Normal	0	25	44	2	71	2	34	36	1	73
	(0%)	(35.2%)	(62%)	(2.8%)		(2.7%)	(46.6%)	(49.4%)	(1.4%)	
Tumor	21	42	40	17	120	45	73	32	11	161
	(17.5%)	(35%)	(33.3%)	(14.2)	*p* < 0.0001	(28%)	(45.3%)	(19.9%)	(6.8%)	*p* < 0.0001

### Somatic mutations of *NKAIN2* are uncommon in prostate cancer

Gene mutation is another mechanism leading to inactivation of TSGs. To detect somatic mutations of *NKAIN2*, the upstream and coding regions of *NKAIN2* were amplified by Fluidigm Access Array and then analyzed using next-generation sequencing. We processed 60 prostate cancer samples, of which 52 had paired adjacent normal samples, five prostate cancer cell lines (PC-3, 22RV1, DU145, VCaP and LNCaP) and two immortalized prostate epithelial cell lines PNT1a and PNT2-C2. More than 100× depth was generated for all *NKAIN2* exon regions except exon 10 and the promoter, for which depth around 50× was achieved. We identified six mutations, including three missense and three synonymous, in four clinical prostate cancer cases (Table 2). No mutations were detected in any cell line.

**Table 2:** Somatic mutations in *NKAIN2* identified by promoter and exon sequencing

Mutation	Predicted function	cDNA position	Coding position	aa position	aa change	Effect	sample
chr6_124125363_C|T	coding	78	18	6	G > G	Synonymous	T70
chr6_124604186_T|C	coding	150	90	30	L > L	Synonymous	T95
chr6_124979372_G|A	coding	374	314	105	R > Q	missense	T70
chr6_124979445_A|G	coding	447	387	129	E > E	Synonymous	T95
chr6_125139554_A|G	coding	617	557	186	D > G	missense	T67
chr6_124604161_T|C	coding	125	65	22	L > P	missense	T83

Abbreviations: aa: amino acid.

### Promoter methylation changes in prostate cancer

**Figure 3:**
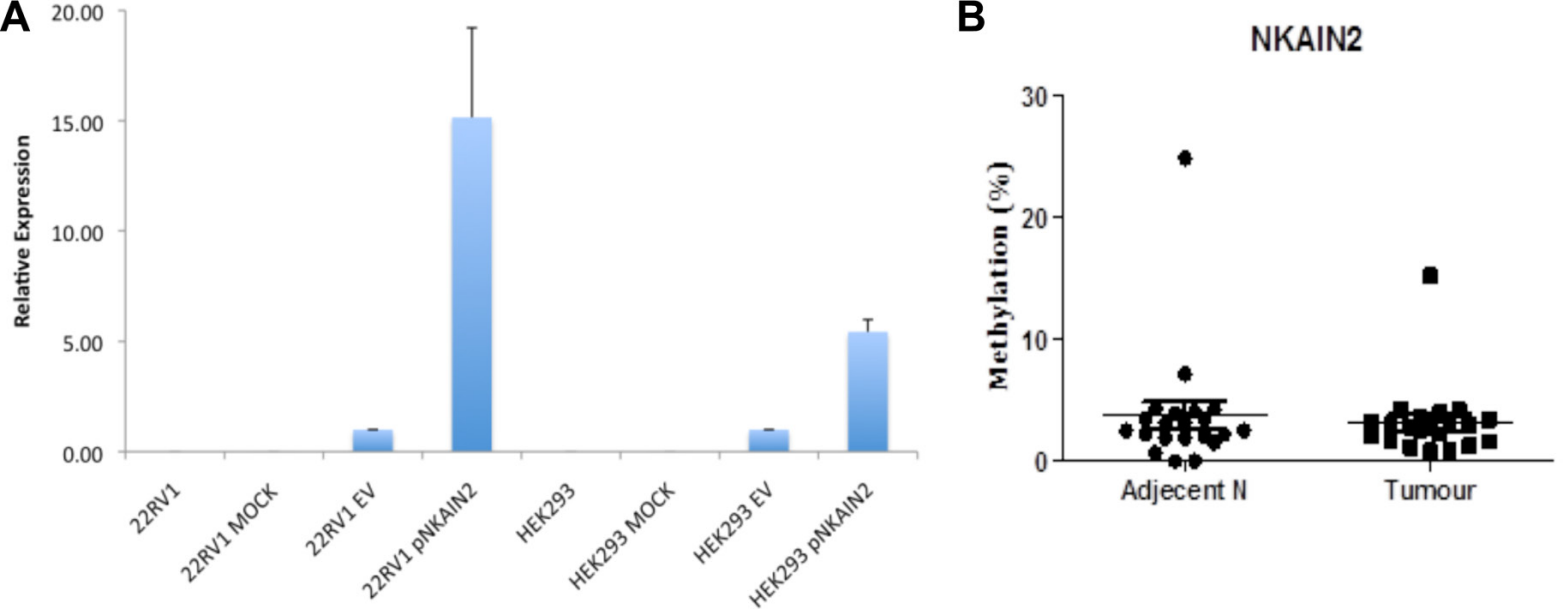
*NKAIN2* promoter activity and DNA methylation analysis (**A**) The activity of the putative 251 bp *NKAIN2* promoter detected by luciferase reporter assay in 22RV1 prostate cancer and HEK293 kidney cell lines. For each cell line, luciferase activity was normalized to cells transfected with the empty reporter construct. (**B**). The percentage of methylated DNA in the exon 1 CpG island in 21 prostate cancer and adjacent normal tissues.

To further investigate if promoter methylation suppresses *NKAIN2* expression, we identified two CpG islands of 470 bp and 698 bp located from −1155 to +61 of *NKAIN2.* A 251 bp promoter residing within these CpG islands was predicted using Proscan and its activity was confirmed using luciferase report gene assay in 22RV1 and HEK293 cells (Figure 3A). Consequently, we selected the promoter region and the CpG island within exon 1 for DNA methylation analysis by pyrosequencing 21 paired cancer and adjacent non-malignant samples. We detected very low level of methylation in the exon 1 CpG sites with no difference between tumor and normal tissues (*p* = 0.8)(Figure 3B). We failed to sequence the promoter region, potentially due to its very high GC content.

### Knockdown of *NKAIN2* increases cell proliferation, migration and invasion and decreases apoptosis of prostate cancer cells

**Figure 4:**
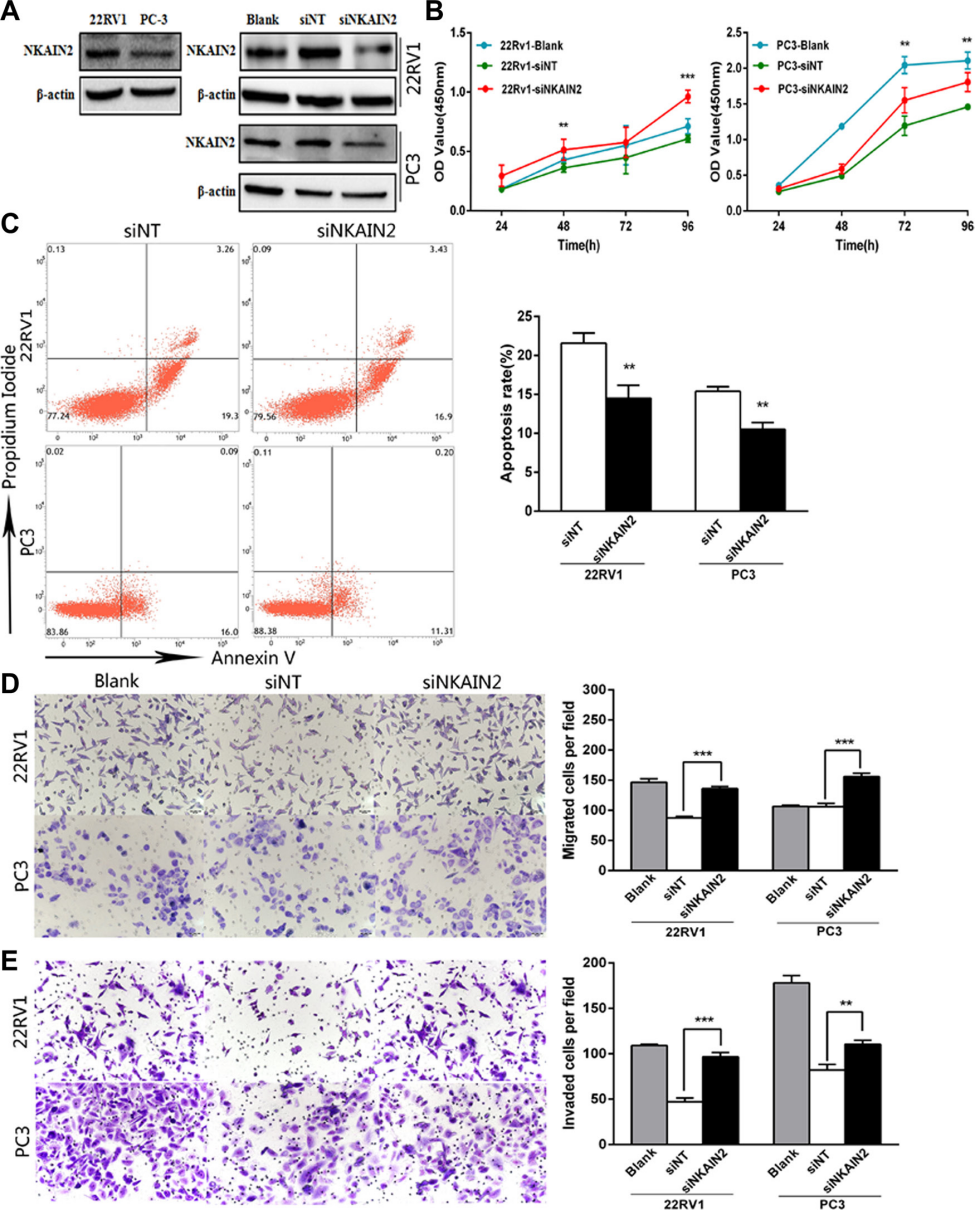
The effect of NKAIN2 knockdown in 22RV1 and PC-3 cells (**A**) The effects of NKAIN2 knockdown confirmed by western blotting. NT: nontargeting siRNA. (**B**) Knockdown of NKAIN2 increased cell growth rate as determined by CCK-8 assay. (**C**) Representative FACS plots analysis showing reduced cell apoptosis rate by NKAIN2 knockdown. (**D**) Transwell migration and (**E**) transwell invasion assays showed that NKAIN2 knockdown promoted prostate cancer cell migration and invasion. Data represent three independent experiments. *p < 0.05, **p < 0.01, ***p < 0.001.

siRNA knockdown of *NKAIN2* was employed to explore the TSG role of NKAIN2 and its potential cellular function in 22RV1 and PC3 prostate cancer cells. NKAIN2 expression as determined by Western blotting was greater in 22RV1 than in PC3 cells, which could be explained by the deletion of *NKAIN2* in PC3 but not 22RV1 cells. Knockdown by siRNA decreased the expression levels of NKAIN2 in 22RV1 and PC3 cells compared to non-targeting siRNA transfected control cells (Figure 4A). This significantly enhanced the growth of 22RV1 and PC3 cells compared to the non-targeting siRNA transfected cells (Figure 4B). To investigate if this increased cell growth correlated with decreased cell death, we analyzed the effect of *NKAIN2* knockdown on apoptosis by flow cytometry analysis and found that apoptosis was inhibited 24 h after knockdown of *NKAIN2* (Figure 4C). Furthermore, the migration and invasion analyses showed that the migration and invasion abilities of 22RV1 and PC3 cells transfected with *NKAIN2* siRNA significantly increased as compared to non-targeting siRNA transfected cells (Figure 4D and 4E).

### Overexpression of *NKAIN2* decreases prostate cancer cell proliferation, migration and invasion and increases apoptosis

**Figure 5:**
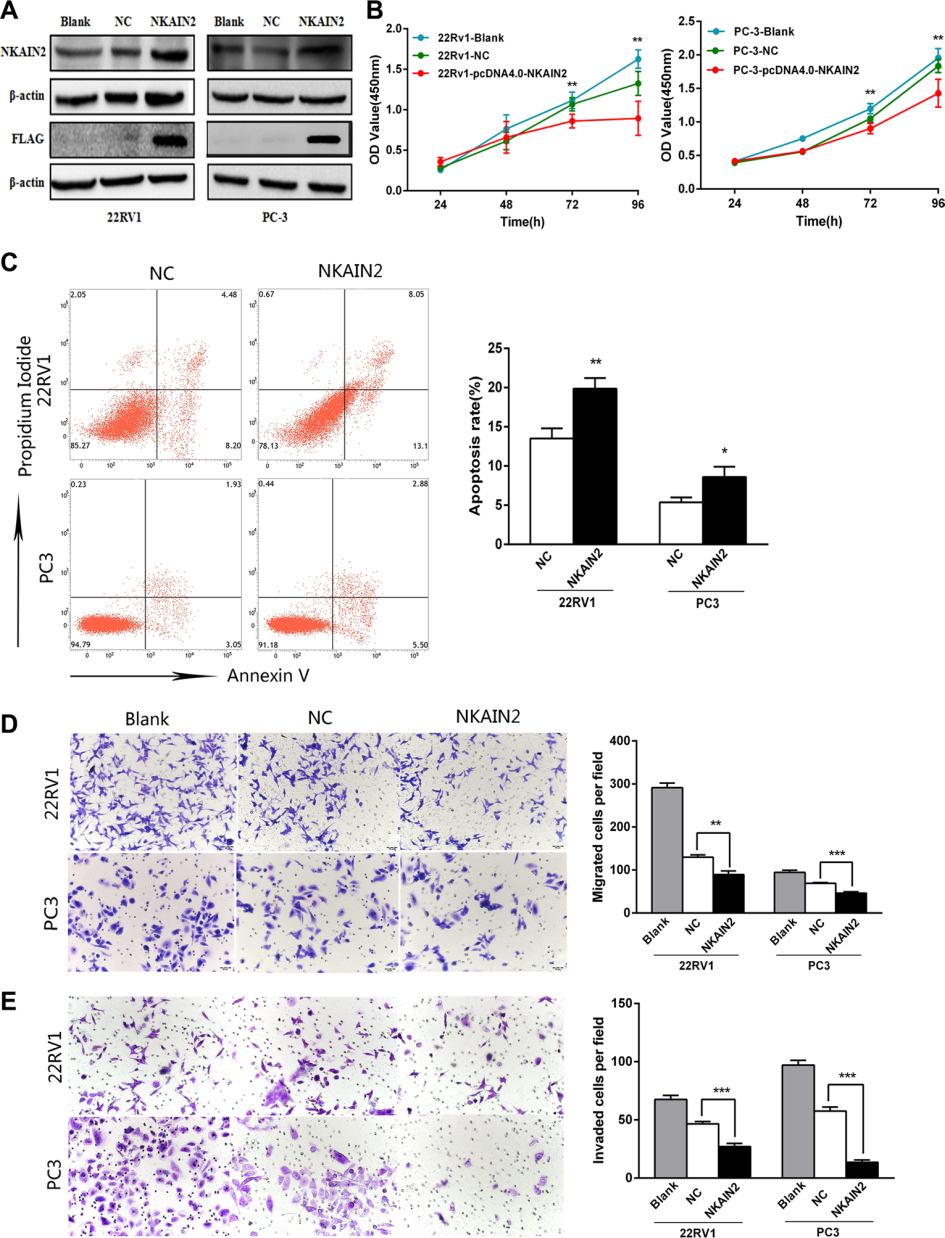
The effect of NKAIN2 overexpression in 22RV1 and PC-3 cells (**A**) The expression of NKAIN2 analyzed by western blotting, NC: negative control. (**B**) Cell growth significantly inhibited by NKAIN2 overexpression as determined by the CCK-8 analysis. (**C**) Representative FACS plots analysis showing increased cell apoptosis rate by overexpression of NKAIN2. (**D**) Transwell migration and (**E**) transwell invasion assays showed that overexpression of NKAIN2 significantly inhibited prostate cancer cell migration and invasion. Error bars represent mean ± SD from three independent experiments. *p < 0.05, **p < 0.01, ***p < 0.001.

To provide more evidence for the tumor suppressor role of *NKAIN2,* 22RV1 and PC3 cells were transiently overexpressed with the *NKAIN2* expressing pcDNA4.0-*NKAIN2*-Flag plasmid. NKAIN2 overexpression was confirmed by Western blot analysis using both anti-NKAIN*2* and anti-FLAG antibodies (Figure 5A). Overexpression of NKAIN2 significantly decreased growth and increased apoptosis in 22RV1 and PC3 cells compared to cells transfected with empty vector (Figure 5B and 5C). Using the transwell assay with and without matrigel, we found that cell invasion and migration also significantly decreased compared to the negative control transfected cells (Figure 5D and 5E).

## DISCUSSION

Deletion of the 6q region, containing several putative TSGs, is a frequent event in human tumors [9, 10–12]. Our recent SNP array analysis revealed that genomic truncations are a common mechanism disrupting TSGs in prostate cancer and *NKAIN2* gene is more frequently detected in prostate cancers from Chinese than UK men. Evidence from this study indicates that *NKAIN2* is a putative TSG found within the recurrently deleted 6q chromosomal region. In addition to our data showing that *NKAIN2* is commonly inactivated by chromosome alterations in prostate cancer, low mRNA expression of *NKAIN2* has been reported in castration-resistant prostate cancer [13]. In neurofibromas, sub-microscopic deletions containing *NKAIN2* were detected by microarray in 5 of 9 samples [12]. Subsequent mining of Oncomine and Gene Expression Omnibus databases revealed under-expression of *NKAIN2* in human brain, central nerve system (http://www.oncomine.org/resource/login) and ovarian (http://www.ncbi.nlm.nih.gov/sites/GDSbrowser?acc=GDS3592) cancers compared to corresponding normal controls. These findings support the tumor suppressor role of *NKAIN2* not only in prostate cancer development and progression, but also in other human malignancies.

*NKAIN2* was found fused to *SUSP1* (SUMO-1-specific protease), creating a *SUSP1:TCBA1* in a T-cell lymphoblastic lymphoma cell line HT-1 [14]. While the oncogenic function of this chimeric gene has not been recognized, a copy number gain and increased expression of *NKAIN2* were found in a study of neuroblastoma [15], suggesting that *NKAIN2* can also act as an oncogene. The ability of the same gene to act either as a TSG or an oncogene, depending on the cellular and tissue context, has been observed for a number of cancer associated-genes [16]. However, in prostate cancer, all previous studies and our data provided complementary evidences that *NKAIN2* is a potential TSG.

Loss of TSG function can result from genomic deletion, mutation and promoter DNA methylation. According to results from this study, chromosomal deletion and truncation are the two main genomic mechanisms leading to *NKAIN2* loss of function in prostate cancer, whereas mutation of *NKAIN2* occurs in prostate cancer at a low frequency. Currently, 80 mutations disrupting the *NKIAN2* coding region have been reported in the COSMIC database (http://cancer.sanger.ac.uk/cosmic). From the COSMIC and International Genome Cancer Consortium (http://dcc.icgc.org/) databases, recurrence of *NKAIN2* mutation has been detected in breast (4/1233 samples), endometrioid (4/494), clear cell renal cell (2/878), lung squamous cell (4/531) and bladder (2/327) carcinomas, lung (7/639), esophagus (2/151) and stomach (6/338) adenocarcinomas, as well as skin malignant melanoma (13/526). Although *NKAIN2* mutations were found in many types of cancers, its low frequency is consistent with our data in prostate cancer. No difference between cancer and adjacent normal tissue were identified through our DNA methylation analysis. Since we were unsuccessful to determine the methylation status of the whole *NKAIN2* promoter region, the contribution of DNA methylation to *NKAIN2* expression requires further investigation.

According to this and our previous study [7], genomic truncation of *NKAIN2* gene occurs mainly in Chinese and rarely in UK prostate cancer samples. All chromosomal rearrangements with breakpoints at *NKAIN2* reported to date in other human malignancies, including neurofibromas [12], T-cell lymphoma and leukemia [14], were found in East Asian patients. These data suggest that population differences in genomic polymorphism or certain environmental genotoxic-carcinogenesis factors in the East Asian countries contribute to truncation of *NKAIN2* during tumorigenesis. Chromosomal translocations at *NKAIN2* gene were observed in the European population, including translocations t(1;6)(q32.3;q22.3) and t(2;6)(q24.3;q22.31), which resulted in constitutional inactivation of *NKAIN2*, but they were detected in patients with developmental delay [17] and neurological disorders [18] rather than with malignancy. The mechanisms leading to the preference of *NKAIN2* truncations for tumorigenesis in East Asian populations warrant further investigation.

NKAIN2 (Na^+^/K^+^-transporting ATPase interacting 2) is a transmembrane protein that binds to Na^+^/K^+^-transporting ATPase subunit beta-1 [19]. However, its role and cellular functions in cancer development and/or progression have not been investigated. By overexpression and knockdown of NKAIN2 in prostate cancer cells, we demonstrated that it suppresses cancer cell growth, induces apoptosis and inhibits cell migration and invasion. Na+/K+-ATPase, which maintains ionic homeostasis, recently has also been found to act as an important signal transducer and to form protein-protein interaction scaffold. Therefore, Na+/K+-ATPase is critical for regulating cell growth, differentiation, survival, migration and invasion as well as cell–cell and cell–substrate adhesion in normal and cancer cells [20, 21]. The beta-subunit of Na+/K+-ATPase is vital for the structural and functional maturation of this iron pump enzyme, the transport of the alpha-subunit to the plasma membrane and the membrane location of the enzyme in epithelia cells [21]. Therefore, NKAIN2 may suppress cancer cell growth, migration and invasion, and induce apoptosis by inhibiting Na+/K+-ATPase activity through binding with its beta-subunit. Increased expression and activity of Na+/K+-ATPase has been observed in cancers and these alterations can be induced by carcinogen before tumor development [20, 21]. More interestingly, it has been reported that androgen suppresses the expression of Na+/K+-ATPase beta subunit, which is higher in androgen-independent than androgen-dependent human prostate cancer cell lines and xenografts [22]. Further studies are required to clarify if NKAIN2 represses the function of Na+/K+-ATPase directly or through collaboration with androgen. Nevertheless, since the tumor suppression effects were observed by increasing and decreasing NKAIN2 expression level in the AR negative PC3 cells, NKAIN2 can clearly suppress prostate cancer cell growth and migration in cells without androgen activity. This is consistent with the report that *NKAIN2* was down-regulated in castration resistance prostate cancer samples [13].

While increased activity of Na+/K+-ATPase promotes cancer development, its reduced expression and activity have also been detected in certain human tumors, including renal cell carcinoma, urothelial cancer and different types of cancer cell lines [21], which is in concordance with the biphasic function of *NKAIN2* as either TSG or oncogene in different cell types. As Na+/K+-ATPase enhances tight junctions and cell polarity, its reduced activity or expression in these cancers were proposed to lead to epithelial-mesenchymal transition [23], a cell feature change important for cancer invasion and metastasis. Further investigations are required to understand how NKAIN2 suppresses Na+/K+-ATPase functions and by which molecular pathways *NKAIN2* restrains prostate cancer development and/or progression.

The Na+/K+-ATPase inhibitors, initially used clinically to control heart failures, appeared to take an effect in preventing human cancers, including prostate cancer. Many such inhibitors have consequently been investigated *in vitro* and *in vivo* for their anti-cancer effects as well as cardiotoxicity, and certain inhibitors are currently under clinical trials [20]. However, the anti-cancer activity of Na+/K+-ATPase inhibitors were previously examined without considering its molecular changes in cancer cells, which is required for such targeted therapy. Treatment using these inhibitors and selected exclusively for cancers with elevated Na+/K+-ATPase activity should increase the therapeutic efficiency and reduce cardiotoxicity. We demonstrated that loss of NKAIN2 function occurs in a large proportion of prostate cancer cases, which may lead to overactivity of Na+/K+-ATPase and consequently prostate cancer development and progression. This promotes the development of a genomic or gene expression diagnostic assay with great prospect to identify substantial number of prostate cancer patients suitable for treatment with Na+/K+-ATPase inhibitors. Further investigations of other downstream mechanisms associated with the tumor suppressor activity of NKAIN2 have the potential to identify more molecular targets for the development of novel therapies, which can be used alone or in combination with Na+/K+-ATPase inhibitors.

In conclusion, we found that *NKAIN2* is commonly inactivated in prostate cancer, particularly in the Chinese cases, by genomic deletion and truncation. *NKAIN2* may act as a tumor suppressor involved in inhibition of cancer cell growth, migration and invasion as well as inducement of apoptosis, potentially through suppressing Na+/K+-ATPase or other molecular pathways. Identification of prostate cancer cases with loss of NKAIN2 functions, and treating them with Na+/K+-ATPase inhibitors with or without combination with other therapeutic drugs may provide an efficient therapeutic approach.

## MATERIALS AND METHODS

### Cell lines and tissue specimens

Six human prostate cancer cell lines, PC-3, 22RV1, LNCaP, DU145, VCaP, MDAPCa2b (ATCC, Manassas, VA, USA), two immortalized human prostate epithelial cell lines, PNT1a, PNT2-C2 (obtained from Norman Maitland), and the human embryonic kidney cell line HEK293 (ATCC), were used in this study. All cell lines were authenticated by STR genotype analysis and cultured in DMEM medium with 10% bovine serum.

Ninety six paired tumor and normal fresh frozen prostate cancer clinical samples from China, three TMAs containing 124 UK prostate cancer cases and four TMAs containing 194 Chinese prostate cancer cases were included for this study. Samples were collected with written informed consent of the patients and approved by research ethics committee in UK and local institutional ethical review boards in China.

### FISH analysis

FISH analysis was performed on TMA samples as previously described [4]. We applied two BACs RP11-423J22 (5′ of *NKAIN2*, labeled green) and RP11-510H23 (3′ of *NKAIN2*, labeled red) to detect both genomic truncations and copy number changes of *NKAIN2*. FISH signals were scanned and captured under a 40× lens using an Olympus fluorescent microscope equipped with a CCD camera (Olympus, Tokyo, Japan) on the Applied Imaging Ariol System (Leica, San Jose, CA, USA). Evaluation of the FISH results was performed in a double-blind manner and a minimum of 100 cells with clear hybridization signals were counted per core.

### Mutation analysis by combined fluidigm selected genomic region amplification and next- generation sequencing

The genomic regions containing all 11 exons of *NKAIN2* and a 450 bp potential promoter region were amplified using Fluidigm Access Array (Fluidigm, Cambridge, UK). PCR product from each primer pair was assessed by Agilent Bioanalyser and further processed for next-generation sequencing on an Illumina MiSeq system (Illumina, San Diego, CA). Sequencing data were aligned with Bowtie 2 [24] and variants were called with VarScan 2 [25] using Genome Reference Consortium human build 37 (GRCh37). A mutation was recorded only when the mutant allele frequency was 10 or more.

### Quantitative reverse transcription PCR (qRT- PCR) analysis

Total RNA was extracted from fresh frozen tissues and cell lines using Trizol (Life technologies, Carlsbad, USA) following the manufacturer's protocol. Tumor and matched adjacent normal tissues were macro-dissected to achieve satisfactory purity of cancer cells. The cDNA was synthesized from total RNA using Superscript II (Life technologies). QRT-PCR was performed using TaqMan Master Mix (Life Technologies) and predesigned TaqMan *NKAIN2* expression probes (Hs00902853_m1) and the endogenously expressed GAPDH gene (Hs99999905_ m1) (Life Technology), as previously described [10]. Each sample was performed in triplicate.

### Promoter activity and DNA methylation analysis

The putative promoter region was cloned into a pGL3-basic luciferase reporter construct and transfected with controls into 22RV1 and HEK293 cells using lipofectamine 2000. The luciferase activity was detected using the One-Glo Luciferase Assay System (Promega, Madison, USA). Genomic DNA from cancer and adjacent normal tissue samples was bisulphite-converted with the Epi-Tect Bisulfite kit (Qiagen, Venlo, Netherlands). Two regions, one for the *NKAIN2* promoter region and one for the CpG island located at exon 1 before the translation start site, were biotin-labeled to amplify the bisulphite-converted DNA and subsequently pyrosequenced as previously described [26].

### Generation of NKAIN2 antibody and Western blotting

A monoclonal mouse antibody binding to peptides (TPAPDWAPED, GYQGPQKTSH, HLQLQPMYMS, DLSKETDLIL, SFDFIGGFDS, TSVTPAPDWA, QGPQKTSHLQ, APEDHRYITV, EEEDSFDFIG, SKETDLILTF) of human NKAIN2 was generated through Abmart (Shanghai, China). For Western blotting analysis, cells were harvested and lysed in the RIPA buffer. The expression levels of proteins transferred onto the membranes were detected by antibodies against NKAIN2 (Abmart), the Flag tag (Sigma-Aldrich) and β-actin (Bioword, USA) using the standard Western blotting method [10].

### Immunohistochemistry

Immunohistochemistry was performed on TMA samples as previously described [10]. NKAIN2 expression levels were scored into four categories: negative, weak positive, intermediate positive and strong positive. The final protein expression score of a sample combined the intensity and percentage of positive cells, as previously described [6].

### Plasmid and siRNA transfection

22RV1 and PC3 were seeded in six-well plates (3 × 10^5^ cells/well) (Corning Costar, Cambridge, USA) with RPMI 1640 medium containing 10% FBS, and incubated for 24 hours. Cells were then transfected with relevant plasmids or siRNAs using Lipofectamine 2000 reagent (Life technologies) in Opti-MEM^®^ I Reduced-Serum Medium (Life technologies) according to the manufacturer's protocol. For transient transfection to overexpress *NKAIN2*, cells were transfected with pcDNA4.0-NKAIN2-Flag or with the empty plasmid (Life technologies). For *NKAIN2* knockdown, cells were transfected with *NKAIN2* ON-TARGET plus SMARTpool siRNA or a scramble RNA negative control (Dharmacon, Lafayette, USA).

### Cell proliferation, migration and invasion assays

The proliferation of post-transfected cells was evaluated using a CCK-8 assay (Dojindo, Kumamoto, Japan) according to the manufacturer's protocol. Briefly, cells were seeded in 96-well plates (3 × 10^3^ cells/well) and cultured for 24, 48, 72, 96 hours before adding 10 μl of CCK-8 to each well. After two-hour incubation, the absorbance value of each well was measured using a SpectraMax M5 microplate reader (Molecular Devices, USA) at 450 nm.

The migration and invasion ability of post-transfected cells were measured using transwell chamber (Corning). 3 × 10^4^ cells resuspended in serum-free medium were added to the upper chamber with and without Matrigel (BD Biocoat, Bedford, USA) coating for invasion and migration assays, respectively. Cells were incubated for 24 hours for migration or 48 hours for invasion analysis. Cells on the upper surface of the membrane were removed with a cotton swab. The cells on the lower surface were fixed, stained and counted under a light microscope in five randomly selected fields.

### Flow cytometry analysis

Twenty-four hours after transfection, the cells were washed twice with cold PBS and proceeded using the annexin V FITC apoptosis detection kit I (BD) according to the manufacturer's instructions. Apoptotic cells were detected by flow cytometry on the BD FACSVerse system.

### Statistical analysis

Statistical analyses were performed using the SPSS (version 20.0) software with two tailed tests. Chi square test was used for categorized data, including FISH and immunohistochemistry results. Mann-Whitney *U* test was applied for RNA expression and DNA methylation data. Student's *t-test* was used for functional study data between the positive and control transfected groups after *NKAIN2* overexpression and knockdown in prostate cancer cell lines. *p* < 0.05 was considered to be statistically significant.
